# The Effect of Magnetotherapy on Back Pain Sensitivity and Muscle Tension in Recreational Horses—A Pilot Study

**DOI:** 10.3390/ani16010077

**Published:** 2025-12-26

**Authors:** Ewa Jastrzębska, Dominika Dobbek, Aleksandra Pawłowska

**Affiliations:** Department of Horse Breeding and Riding, University of Warmia and Mazury in Olsztyn, Oczapowskiego 5, 10-719 Olsztyn, Poland

**Keywords:** thermal imaging, magnetotherapy, inflammation reduction, back injuries, horse riding

## Abstract

Back pain in horses is a frequent musculoskeletal issue that affects performance and welfare. Magnetotherapy has been proposed as a complementary, non-invasive treatment to reduce pain and support soft tissue recovery, but studies in horses remain limited. This pilot study aimed to evaluate the effects of low-frequency pulsed magnetic field therapy on horses with hypersensitivity to palpation along the longissimus dorsi muscle. Four recreational horses participated in a 10-session magnetotherapy program, with changes assessed using palpation, neck flexibility tests, heart rate measurements and thermal imaging. Results showed a reduction in pain sensitivity and muscle tension, particularly in the withers, thoracic, lumbar and sacral regions. Heart rate decreased after treatment, which may indicate a relaxing effect. Thermal imaging confirmed that magnetotherapy did not increase surface temperature, confirming its non-thermal nature. No adverse effects or swelling were observed in any of the horses. These findings provide preliminary data from this pilot study, suggesting that magnetotherapy may be a beneficial adjunct in the treatment of back pain in horses, promoting relaxation and pain relief without inducing tissue heating. Further research on larger populations with a negative control group is needed to validate these findings and support broader clinical application.

## Simple Summary

Back pain is a common issue among riding horses and can negatively affect their comfort and performance. Magnetotherapy, as a non-invasive treatment that uses magnetic fields, is believed to reduce pain and help muscles recover. In this pilot study, four recreational horses with back problems (hypersensitivity to palpation) received magnetotherapy treatments. Their responses were measured by evaluating back pain sensitivity through palpation and movement tests and using thermal imaging to assess changes in temperature. The results showed a decrease in back pain and muscle tension after the therapy. The horses were visibly more relaxed during and after the treatment, as evidenced by a reduction in heart rate. Importantly, the therapy did not raise the temperature of the horses’ backs, confirming that it does not generate heat and is safe to use, even with acute, early-stage inflammation. These findings support the use of magnetotherapy as a helpful addition to standard care for horses with back problems. This therapy may improve equine comfort and support their long-term well-being.

## 1. Introduction

Back pain sensitivity frequently occurs among musculoskeletal problems in riding horses, posing a significant challenge to equine comfort, health and performance [1,2,3,4]. It is increasingly emphasized that a saddle not properly adapted to the horse’s conformation and way of moving may represent a significant source of back-related health problems. Improper distribution of pressure, resulting from poor saddle fit, contributes to the development of soft tissue soreness as well as overloading of musculoskeletal structures [5,6,7]. Conventional treatments, including rest, physiotherapy, pharmacological interventions and corrective training, often require prolonged rehabilitation and do not always yield satisfactory outcomes [8]. Therefore, there is a growing interest in complementary therapeutic methods that can support recovery and improve the welfare of equine.

Magnetotherapy, the therapeutic application of static or low-frequency pulsed electromagnetic fields (PEMF), has been increasingly explored in both human and veterinary medicine [2,9,10]. Its mechanism of action is thought to involve modulation of ion exchange at the cellular level, improved microcirculation, enhanced oxygen supply to tissues, stimulation of collagen synthesis and reduction in inflammatory mediators [4,10,11]. Magnetotherapy is applied in the treatment of various equine conditions, including bone fractures and injuries, joint degeneration, soft tissue injuries and the reduction in inflammatory states [12,13,14,15,16,17]. These physiological effects may contribute to accelerated healing of soft tissue injuries, pain relief and overall functional recovery [9].

Despite promising experimental and clinical findings in other species, scientific evidence for the efficacy of magnetotherapy in treating equine back injuries remains limited and requires further validation [2,8].

There is a growing need for non-invasive and practical methods to assess back discomfort in horses, as clinical signs are often subtle and traditional diagnostic procedures are not always feasible in everyday practice. Only a limited number of studies have examined the potential effects of magnetotherapy on equine musculoskeletal conditions and the available evidence remains inconclusive. Therefore, we conducted a pilot study to explore whether low-frequency pulsed magnetotherapy may influence selected indicators of back discomfort, including palpation sensitivity, thermographic patterns and functional responses. The objective of this study was to investigate the effect of magnetotherapy on indicators of back discomfort in recreational horses. We formulated the hypothesis that magnetotherapy would reduce signs of back discomfort, reflected primarily by decreased palpation sensitivity and improved thermographic and functional responses.

## 2. Materials and Methods

The study was conducted on a group of four recreational geldings exhibiting back pain sensitivity. Two of the horses were Silesian, aged 13 and 15 years, and the other two were Polish Sport Horses, aged 13 and 18 years.

All animals were kept in individual boxes but spent several hours daily together in a shared paddock or pasture. They had free access to hay and straw, and concentrated feed was provided three times a day. Routine veterinary and physiotherapy examinations confirmed that all horses were in good general health, with the only finding being pain sensitivity along the longissimus dorsi muscle. During these assessments, direct palpation of the paraspinal muscles, skin stimulation with a brush, use of a specialized roller and observation of posture and body symmetry were employed to identify areas of discomfort. The most likely contributors to this sensitivity were the cumulative workload associated with regular recreational use and the fact that the horses were ridden by multiple riders of varying experience, as they were used in a lesson program and worked several times per week.

Each horse participated in regular recreational riding sessions lasting approximately six hours per week, consisting of 15 min of walk, 20 min of trot, 10 min of canter and a final 15 min of walk. Riding took place outdoors in a sand-based arena under consistent surface conditions.

Before the magnetotherapy intervention, a control trial was conducted over five consecutive days. During this phase, horses participated only in their regular riding sessions, without any additional treatment. All assessments were conducted under the supervision of a trained animal physiotherapist, who was blinded to the treatment (single-blinded design). Pain sensitivity along the longissimus dorsi muscle and neck flexibility were evaluated using the palpation and hard brush tests, with the pain and neck flexibility scales specifically developed by the authors for this study (Table 1 and Table 2). Assessments were performed on both sides of the horse’s body in the following regions: withers, thoracic, lumbar, sacral, including the evaluation of neck rotation.

After completing the control phase, the horses underwent a series of ten magnetotherapy sessions. The treatments were performed using flat applicators from G-Pulse (Equimag GmbH, Darmstadt, Germany). Sessions were conducted around 4:00 p.m., after the horses had returned from the paddock and feeding, to ensure calm and stable environmental conditions. Treatments were given daily for the first five sessions and every other day for the remaining five sessions (Table 3). During each procedure, the horses stood calmly at a hitching post in an isolated area of the stable to minimize distractions.

Pain and muscle tension assessments (palpation and brush tests) were performed immediately before and after each magnetotherapy session. Heart rate was measured at the beginning and end of each magnetotherapy session by palpating the facial artery as it crosses the medial aspect of the mandible at the mandibular notch.

To evaluate surface temperature changes associated with magnetotherapy, thermal images were taken using a FLIR T250 thermal imaging camera (Teledyne FLIR LLC, Wilsonville, OR, USA). Imaging was performed in an indoor riding arena to ensure optimal and stable environmental conditions. Ambient temperature was approximately 20–22 °C, relative humidity was 60–70%, and no significant air currents were detected. Horses were groomed and thermographic imaging was performed at least 40 min after grooming, with no ridden exercise on imaging days. Photographs were taken from a ladder at a height of approximately 3 m to visualize the dorsal midline.

Thermal data were analyzed using FLIR Tools software (version 6.4). An elliptical region of interest (ROI) was placed along the dorsal midline of the horse’s back, covering the thoracolumbar region corresponding to the longissimus dorsi muscle treated with magnetotherapy. Within this ROI average, minimum and maximum temperatures were calculated and compared between baseline (before treatment) and after the 1st, 5th and 10th magnetotherapy sessions.

Statistical analyses were conducted using Statistica 13.3 (TIBCO Software Inc., Palo Alto, CA, USA, 2017). The Shapiro–Wilk and Lilliefors tests were used to assess data normality. The Wilcoxon signed-rank test evaluated changes before and after riding and magnetotherapy sessions. The Kruskal–Wallis test with post hoc analysis compared differences between horses. Paired Student’s *t*-test and one-way ANOVA with Duncan’s multiple range test were used to analyze thermographic data. Statistical significance was set at *p* < 0.05. The statistical analyses used here are exploratory and intended to illustrate preliminary trends.

## 3. Results

### 3.1. The Effect of Magnetotherapy on Back Tension and Pain Sensitivity in Horses

**Figure 1:**
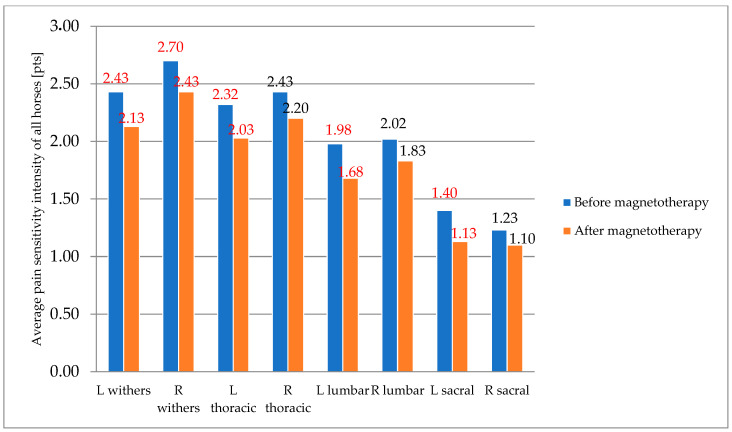
Assessment of pain sensitivity intensity in the studied body regions of horses subjected to magnetotherapy. Mean values are shown for descriptive purposes only. Significant differences (marked in red) indicate *p* < 0.05 based on exploratory analyses.

A significant change (*p* < 0.05) was observed in the assessment of withers’ pain sensitivity, with a reduction in both tension and pain sensitivity. Tension decreased on both sides, and all horses showed a greater degree of relaxation. In the thoracic segment, tension was significantly lower only on the left side, while a slight improvement was noted on the right side, although it was not significant (*p* > 0.05). The lumbar segment, due to its high mobility, is particularly prone to overload, leading to significant tension. A significant reduction in pain sensitivity was observed on the left side of the horses following magnetotherapy treatment. Additionally, a significant (*p* < 0.05) decrease in pain sensitivity in the sacral region of the spine was noted on the left side, which may be attributed to the similar tension on that side in both the thoracic and lumbar regions (Figure 1).

### 3.2. Back Pain Sensitivity in the Hard Brush Test in Horses Subjected to Magnetotherapy

**Figure 2:**
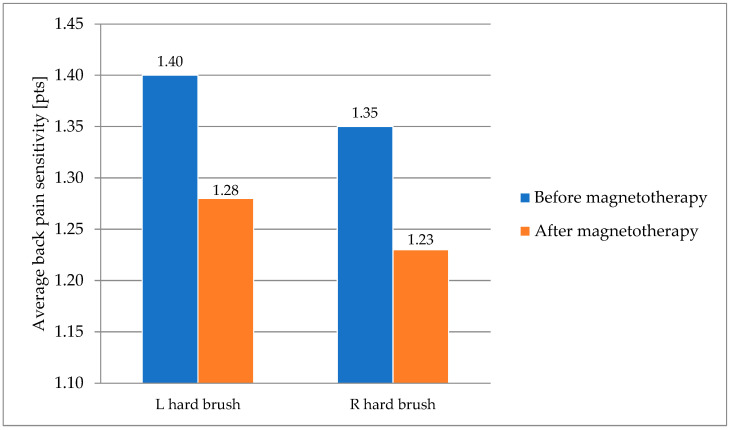
The hard brush test in horses subjected to magnetotherapy. Mean values are shown for descriptive purposes only.

In the hard brush test, no significant (*p* > 0.05) differences were observed in the horses’ behavior after undergoing magnetotherapy. A slight decrease in tension was noted on both the right and left sides (Figure 2).

### 3.3. Assessment of Neck Flexibility in Horses Subjected to Magnetotherapy

**Figure 3:**
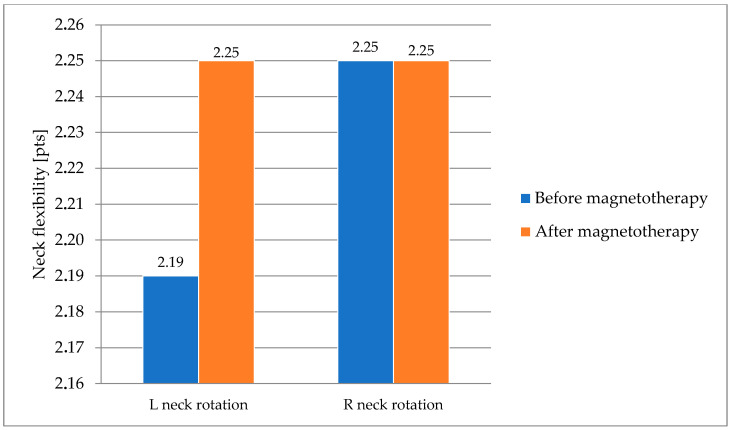
Neck flexibility in horses subjected to magnetotherapy. Mean values are shown for descriptive purposes only.

No significant differences were observed in the neck flexion of the horses following magnetotherapy (Figure 3).

### 3.4. Heart Rate and Thermal Imaging

**Figure 4:**
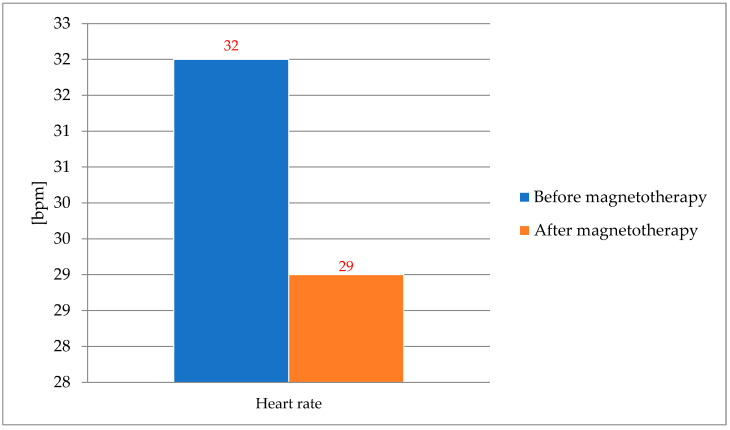
The average heart rate of horses subjected to magnetotherapy. Mean values are shown for descriptive purposes only. Significant differences (marked in red) indicate *p* < 0.05 based on exploratory analyses.

A significant (*p* < 0.05) decrease in the horses’ heart rate was noted during each magnetotherapy treatment (Figure 4).

A comparison of average temperatures depending on the number of magnetotherapy sessions performed did not show any significant changes. The temperature of the horses’ backs did not vary according to the progression of the magnetotherapy treatments (Table 4).

## 4. Discussion

In the current study, the effect of magnetotherapy on changes in back tension and pain sensitivity in horses was evaluated. A significant improvement in back pain sensitivity and muscle tension was observed in response to magnetotherapy, particularly in the withers, thoracic and lumbar regions on the left side of the body, which initially exhibited greater tenderness. These findings suggest a reduction in local pain and muscle tension in the regions located directly beneath the magnetic blanket applicator. The improvement was therefore likely related to the local influence of the pulsed magnetic field rather than a generalized systemic effect. However, no significant (*p* > 0.05) changes were detected in neck flexion or in the horses’ responses to the hard brush test. This lack of effect on cervical mobility can be explained by the fact that the magnetic blanket applicator did not cover the neck region and therefore had limited direct impact on the neck muscles. Moreover, neck flexion may not have been initially restricted by thoracolumbar discomfort but rather represented a compensatory or independent function. Similarly, the hard brush test, which assesses generalized tactile sensitivity, may not have been sufficiently specific to detect subtle changes resulting from the treatment applied to the back. These observations highlight the need for more targeted functional assessments in future studies to evaluate region-specific responses.

Reduction in pain symptoms in response to magnetotherapy was confirmed in the study performed by Jutrzenka-Jesion 2015 [18]. A significant decrease in pain in humans was observed, with the analgesic effect persisting up to two weeks after the magnetotherapy sessions. The study also analyzed the influence of therapy on body posture—each patient was diagnosed with an asymmetry that caused pain. After magnetotherapy, a significant improvement in posture and reduction in pain related to it were noted. Palpation results during the study showed a significant improvement in muscle tone after the use of magnetotherapy. These findings support the thesis that magnetic field treatments have a beneficial effect on the body.

Swelling in the withers area is a common injury observed in horses using poorly fitted saddles. The impact of magnetotherapy on swelling levels was studied [19]. A reduction in swelling was observed in both treatment groups. Most horses responded very positively to the therapy, although a small portion of individuals in each group showed no response to the magnetotherapy. In the present study, no clinically significant swelling was observed in any of the horses following magnetotherapy treatments. All horses had been evaluated by a veterinarian and a physiotherapist prior to the study and the only finding was localized pain sensitivity along the longissimus dorsi muscle.

The effect of magnetotherapy on reducing post-exercise muscle acidosis in humans was studied. In the active group, 10 min magnetotherapy applications were performed on the most painful area immediately after exercise, and then again 24 and 48 h after the end of activity. The control group in that study followed the same procedure using a sham device. The results confirmed that magnetotherapy in the active group alleviated physiological problems caused by muscle acidosis [20].

The results of the authors’ own research indicate that the use of magnetotherapy in horses had a positive effect on reducing pain in various sections of the spine. Horses undergoing the treatments showed visibly less discomfort and reduced muscle tension in the back area, which may indicate the analgesic and relaxing effects of this form of therapy.

A study by Rindler et al. 2014 [21] demonstrated that magnetotherapy is a non-thermal procedure, which allows its safe use even in the early stages of inflammation. In the experiment, a thermal imaging camera was used to assess changes in surface body temperature after therapy. One group of horses was treated with a flat magnetic field applicator placed on the back, while the control group received a placebo in the form of a blanket. The analysis of the thermographic images confirmed that magnetotherapy treatments do not cause an increase in body temperature, indicating the absence of a heating effect and confirming the safety of using this method in the early phase of injury or inflammation [21].

Similar results were obtained in a study examining changes in muscle blood flow, skin temperature, pain sensitivity and behavior in healthy horses [22]. Both magnetic and placebo blankets caused an increase in skin temperature during treatment, although no significant differences between the groups were found. The temperature changes were therefore attributed to the insulating effect of the blanket itself rather than the magnetic field.

In the current study, similar thermographic measurements were taken on selected days of magnetotherapy and the temperatures were compared with those from the following treatment day. During the study, ambient temperature and humidity were also monitored, showing correlations with the recorded results. No significant increase in tissue temperature was observed, indicating that under the conditions and parameters applied, magnetotherapy may act primarily through non-thermal mechanisms. A potential relaxation effect was inferred from slight decrease in the animals’ heart rate and palpation assessment. The reduction in heart rate observed after magnetotherapy must be interpreted with caution, as the absence of a negative control group does not allow us to exclude alternative explanations such as habituation, handling-related relaxation or natural recovery unrelated to treatment. These observations suggest that, despite the absence of a measurable thermal effect, magnetotherapy may support relaxation and regenerative processes through non-thermal mechanisms, although no direct physiological, biochemical or EMG measurements were performed in this study to confirm these effects. This aligns with the principle that contemporary physiotherapeutic methods can relieve inflammation without the thermal effect of therapy.

In future studies, it would be valuable to include a larger number of horses to make the results more representative and allow for generalization to a broader population. Moreover, the use of a separate control or placebo group would enable a clearer assessment of the actual effects of magnetotherapy. Long-term observations, involving regular magnetotherapy sessions over several months, would allow for evaluation of the durability of the treatment effects. The introduction of objective measurement tools could reduce the subjectivity in assessing pain and muscle tension. The scales used in manuscript are not formally validated, which limits the objectivity and comparability of results; however, they were designed to provide preliminary data to guide future studies and potential development of validated assessment tools. Studies involving different groups of horses—including sport, racing, older horses or those with musculoskeletal issues—would help determine whether the effects of magnetotherapy are universal or dependent on the specific characteristics of the population.

## 5. Conclusions

This pilot study suggests that low-frequency pulsed magnetotherapy may reduce muscle tension and back sensitivity in recreational horses, particularly in areas that initially showed the greatest discomfort. The therapy was also associated with a decrease in heart rate, which may indicate a calming effect. Changes observed in thermographic patterns were noted, but the underlying mechanisms cannot be determined from this study. Overall, these preliminary findings indicate that magnetotherapy could be a promising approach to support soft tissue relaxation and back comfort in horses undergoing intensive riding, while further studies with larger sample sizes and control groups are needed to confirm these effects.

## Figures and Tables

**Table 1:** Scale for assessing pain sensitivity in horses.

Pain Scale	Symptoms
1	The horse stands calmly, is relaxed, has normal muscle tension, does not react to palpation and has a calm expression;
2	The horse is alert, aware of its surroundings and responds to palpation by slightly bending its body;
3	A very strong pain reaction—the horse moves away from palpation and does not allow approach again.

**Table 2:** Scale for assessing lateral neck flexion.

Flexion Scale	Range of Lateral Neck Flexion
3	The flexion does not cause the horse any difficulty; it performs a full flexion on the first attempt, reaching its muzzle to the ribs;
2	The flexion does not cause the horse any difficulty; it performs a full flexion on the second or third attempt, reaching its muzzle to the shoulder;
1	The flexion is difficult for the horse; the movement is very limited or the horse refuses to perform it.

**Table 3:** Therapy parameters used in the study.

Program	Program Features	Number of Sessions
P1	2 mT/4 Hz/30 min	3 days
P2	5 mT/12 Hz/30 min	2 days
P3	8 mT/25 Hz/30 min	5 days

**Table 4:** Comparison of the average temperature in the dorsal region of horses before and after magnetotherapy treatment.

Treatment Number	Average Temperature	
	Before Treatment	After Treatment
1	21.05	20.23
5	20.58	20.20
10	17.05	16.98

## Data Availability

The original contributions presented in this study are included in the article. Further inquiries can be directed to the corresponding author.
